# Neurl4 contributes to germ cell formation and integrity in *Drosophila*

**DOI:** 10.1242/bio.012351

**Published:** 2015-06-26

**Authors:** Jennifer Jones, Paul M. Macdonald

**Affiliations:** Department of Molecular Biosciences, Institute for Cellular and Molecular Biology, The University of Texas at Austin, Austin, TX 78712-0159, USA

**Keywords:** Primordial germ cells, *Neurl4*, *CP110*, *Oskar*

## Abstract

Primordial germ cells (PGCs) form at the posterior pole of the *Drosophila* embryo, and then migrate to their final destination in the gonad where they will produce eggs or sperm. Studies of the different stages in this process, including assembly of germ plasm in the oocyte during oogenesis, specification of a subset of syncytial embryonic nuclei as PGCs, and migration, have been informed by genetic analyses. Mutants have defined steps in the process, and the identities of the affected genes have suggested biochemical mechanisms. Here we describe a novel PGC phenotype. When *Neurl4* activity is reduced, newly formed PGCs frequently adopt irregular shapes and appear to bud off vesicles. PGC number is also reduced, an effect exacerbated by a separate role for *Neurl4* in germ plasm formation during oogenesis. Like its mammalian homolog, *Drosophila* Neurl4 protein is concentrated in centrosomes and downregulates centrosomal protein CP110. Reducing CP110 activity suppresses the abnormal PGC morphology of *Neurl4* mutants. These results extend prior analyses of *Neurl4* in cultured cells, revealing a heightened requirement for *Neurl4* in germ-line cells in *Drosophila*.

## INTRODUCTION

During embryogenesis a subset of cells become specified as primordial germ cells (PGCs), which will later produce eggs and sperm (Richardson and Lehmann, 2010). In some animals PGC formation is dependent on germ plasm, a specialized cytoplasm containing maternal mRNAs and proteins (Saffman and Lasko, 1999). In *Drosophila*, assembly of germ plasm begins during oogenesis, with localization of *oskar* (*osk*) mRNA to the posterior pole of the oocyte. Local translation of Osk protein then initiates recruitment of maternal mRNAs and proteins (Mahowald, 2001). During the early stages of embryogenesis, in which the nuclei divide without cell division, the germ plasm persists at the posterior pole of the embryo (Mahowald, 1971). At this location a small number of nuclei are the first to cellularize, and inclusion of the germ plasm specifies them as PGCs (Illmensee and Mahowald, 1974; Lehmann and Nüsslein-Volhard, 1986; Ephrussi and Lehmann, 1992). The PGCs are morphologically and behaviorally distinct from the somatic cells, which form after several more rounds of nuclear division. Whereas somatic cells at this developmental stage have a consistent polarity and elongate shape, the PGCs are round and without consistent polarity. As embryogenesis proceeds, the PGCs become polarized and initiate migration to the developing gonad (Richardson and Lehmann, 2010).

Mutants have been identified that affect different steps in PGC formation and behavior. A class of maternal effect mutants reduce or eliminate germ plasm assembly, resulting in embryos with few or no PGCs (Williamson and Lehmann, 1996; Mahowald, 2001). Other mutants retain normal germ plasm, but the PGCs fail to form (Jongens et al., 1992; Robertson et al., 1999). A number of genes are required for PGC migration: the PGCs form normally in the mutant embryos, but are defective in one or more of the multiple steps in migration (Richardson and Lehmann, 2010).

Here we report a novel, dominant phenotype affecting PGCs. When the activity of *Neurl4* is reduced, the normally spherical PGCs of blastoderm stage embryos frequently adopt irregular shapes and appear to bud off vesicles. PGC number is reduced, presumably because of the defects associated with abnormal PGC morphology, but also because of a requirement for *Neurl4* in the initial steps of germ plasm assembly during oogenesis. Just as shown for mammalian Neurl4 protein in cultured cells (Li et al., 2012; Al-Hakim et al., 2012), *Drosophila* Neurl4 protein is concentrated in centrosomes and acts to downregulate centrosomal protein CP110. These results reveal a germ cell-specific role for *Neurl4*. The same Neurl4/CP110 biochemical pathway that prevents formation of ectopic microtubule organizing centers in mammalian cells also affects germ cell morphology.

## RESULTS

### Reduction of maternal *Neurl4* activity affects PGC morphology

**Fig. 1.**
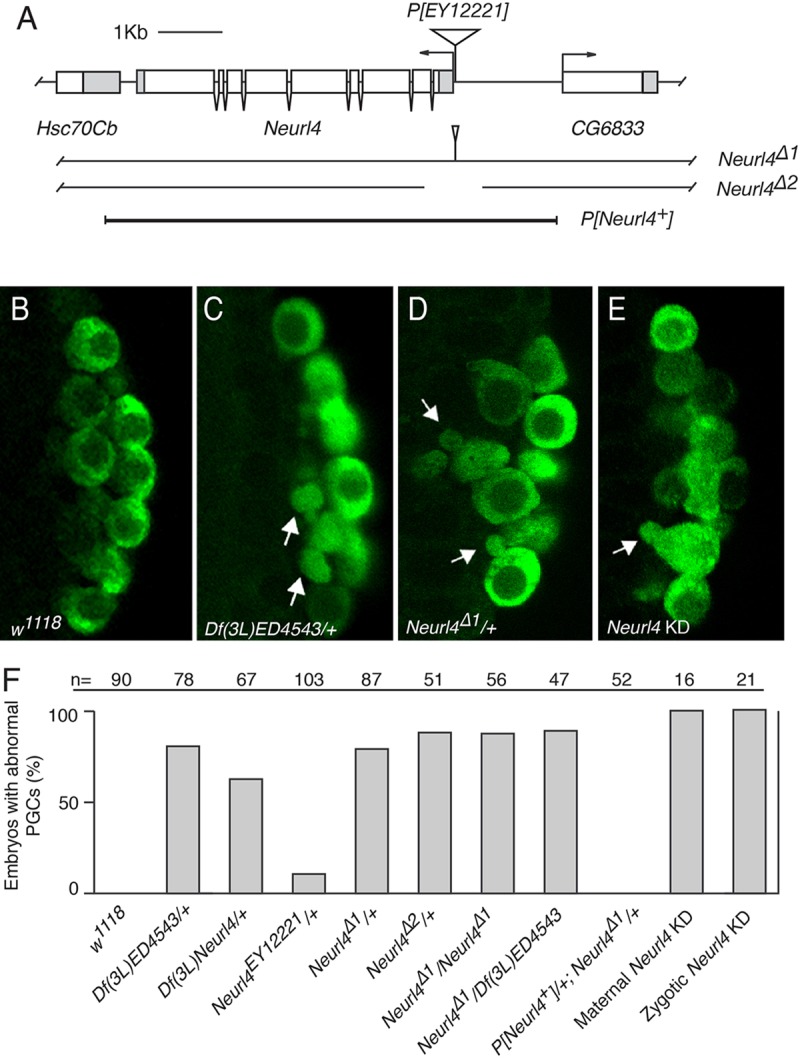
**Abnormal PGCs result from reduced Neurl4 maternal gene dosage.** (A) *Neurl4* gene and mutations. The *Neurl4^EY12221^* insertion is located 4 bp before the predicted transcription start site for *Neurl4*. In *Neurl4*^Δ*1*^, a small portion of the P-element remains, while in *Neurl4*^Δ*2*^, the first exon and a portion of the second exon are deleted, as well as 409 bp upstream of the transcription start site, as indicated by the break in the line. The genomic rescue construct, *P[Neurl4^+^]*, contains the region depicted by the thick line. (B-E) Stage 5 embryos (posterior region) with PGCs detected by Vas staining (green). Maternal genotypes for the embryos are: (B) *w^1118^* (wild type); (C) *Df(3L)ED4543/+*; (D) *Neurl4*^Δ*1*^*/+*; (E) *P{TRiP.GL01219]attP40/+; P{matalpha4-GAL-VP16}V37/+* (*Neurl4* KD). Arrows indicate examples of misshapen PGCs. (F) Frequency of embryos showing abnormal PGCs. Each embryo scored as abnormal had multiple defective PGCs similar to those shown in panels C-E. Embryos scored as wild type had no abnormal PGCs. We never observed embryos with 1–2 abnormal PGCs. The maternal genotypes are shown. For the maternal KD, mothers had *P{TRiP.GL01219}attP40* and *matalpha4-GAL-VP16*. For the zygotic KD, females with *matalpha4-GAL-VP16* were crossed to males with *P{TRiP.GL01219}attP40*. *n* values are for the number of embryos scored.

Newly formed PGCs of stage 4 and 5 embryos (syncytial blastoderm and cellular blastoderm, respectively) can be identified by the presence of Vas protein. At this stage the PGCs are predominantly spherical (Fig. 1B). By contrast, similarly staged embryos from mothers heterozygous for deficiencies (Dfs) that remove the 70A3 region of chromosome 3 displayed a dominant phenotype in which most embryos (60–80%; Fig. 1F) included multiple PGCs with strikingly abnormal morphology (Fig. 1C). The cells had an irregular shape, often with small protrusions. In some cases the protrusions appeared to pinch off from the larger part of the cell: examples of small Vas-positive vesicles were found linked with a larger cell by a fine stalk, or nearby but not detectably connected. Often, the PGCs were not as tightly coalesced as for wild type. Instead of the one or two layers of closely packed PGCs, gaps sometimes appeared between the PGCs, and the layered organization could be disrupted (e.g. Fig. 1D, see also later figures).

Several lines of evidence showed that these dominant PGC defects were due to reduced activity of *CG6451*, the *Drosophila* homolog of mammalian *Neurl4* (*CG6451* is incorrectly annotated as *bluestreak (blue)*, and for consistency we renamed the gene as *Neurl4*; see Materials and Methods). First, a viable mutant with a transposon insertion immediately 5′ to the *Neurl4* transcription unit, *P[EY12221]* (hereafter referred to as *Neurl4^EY12221^*) also displayed the dominant PGC phenotype, although the frequency was significantly lower than for the Dfs (Fig. 1F). Excision of this transposon reverted the phenotype. Second, the *Neurl4*^Δ*1*^ and *Neurl4*^Δ*2*^ mutants (Fig. 1A), obtained by imprecise excision of *Neurl4^EY12221^*, both showed the phenotype (Fig. 1D and F). For these mutants the defects were similar in strength to the Dfs, with abnormal PGCs in well over half of the embryos from heterozygous mothers. Third, the PGC phenotype was fully rescued by a transgene bearing a segment of genomic DNA including the entire *Neurl4* transcription unit and flanking intergenic regions, but neither of the adjacent genes (Fig. 1F). Thus, the abnormal PGC morphology of these mutants was due to reduced *Neurl4* activity.

The frequency of PGC defects was similar in progeny embryos of *Neurl4*^Δ*1*^/+ females crossed with either *Neurl4*^Δ*1*^/+ or wild type males, demonstrating that the PGC phenotype was independent of zygotic genotype. Embryos from wild type females crossed to *Neurl4*^Δ*1*^/*Neurl4*^Δ*1*^ males did not show the PGC phenotype. Therefore, reduced *Neurl4* activity from the mother was the cause of the PGC phenotype. For simplicity, we refer to embryos from the mutant mothers as *Neurl4* mutant embryos.

Although *Neurl4* mutants had a maternal effect on PGCs, this property revealed the source of the required mRNA or protein, but not whether this phenotype was due to reduced *Neurl4* action in the developing oocyte or in the embryo. To distinguish between these options a knock down (KD) approach was used, relying on a transgene from the Transgenic RNAi Project (TRiP) (Ni et al., 2011). This transgene expresses, under UAS/GAL4 transcriptional control, a short helical RNA (shRNA) that targets the *Neurl4* mRNA for degradation. For expression we used a GAL4 driver which is active in the female germ line. In an initial test to determine if the KD was effective and produced a phenotype similar to that of the *Neurl4* mutants, the *Neurl4* KD was performed during oogenesis (i.e. the females had both the driver and the *Neurl4* TRiP transgene). In this situation the PGC phenotype appeared and was fully penetrant, affecting all of the progeny embryos (Fig. 1E,F).

Having established that the *Neurl4* KD was effective, we asked if the gene product is required in the embryo. To limit the *Neurl4* KD to the embryo, females with the GAL4 driver were crossed to males with the *Neurl4* TRiP transgene. Embryos from this cross have maternally-loaded GAL4, which directs expression of the *Neurl4* shRNA once zygotic transcription commences [as early as nuclear division cycle 8, prior to PGC formation at cycle 10 (Pritchard and Schubiger, 1996)]. Notably, the PGC defects were observed in all of the embryos scored. Therefore, we conclude that maternally-provided *Neurl4* was required in the embryo for normal PGC morphology. Furthermore, whatever Neurl4 protein was provided maternally was not sufficient for normal PGC morphology. In mammalian cells Neurl4 is downregulated by proteasome-mediated degradation, following ubiquitylation by HERC2, a HECT E3 ligase (Al-Hakim et al., 2012). If *Drosophila* Neurl4 is subject to the same regulation, continual synthesis of the protein may be required to maintain its level and maternally-supplied protein would not persist.

**Fig. 2.**
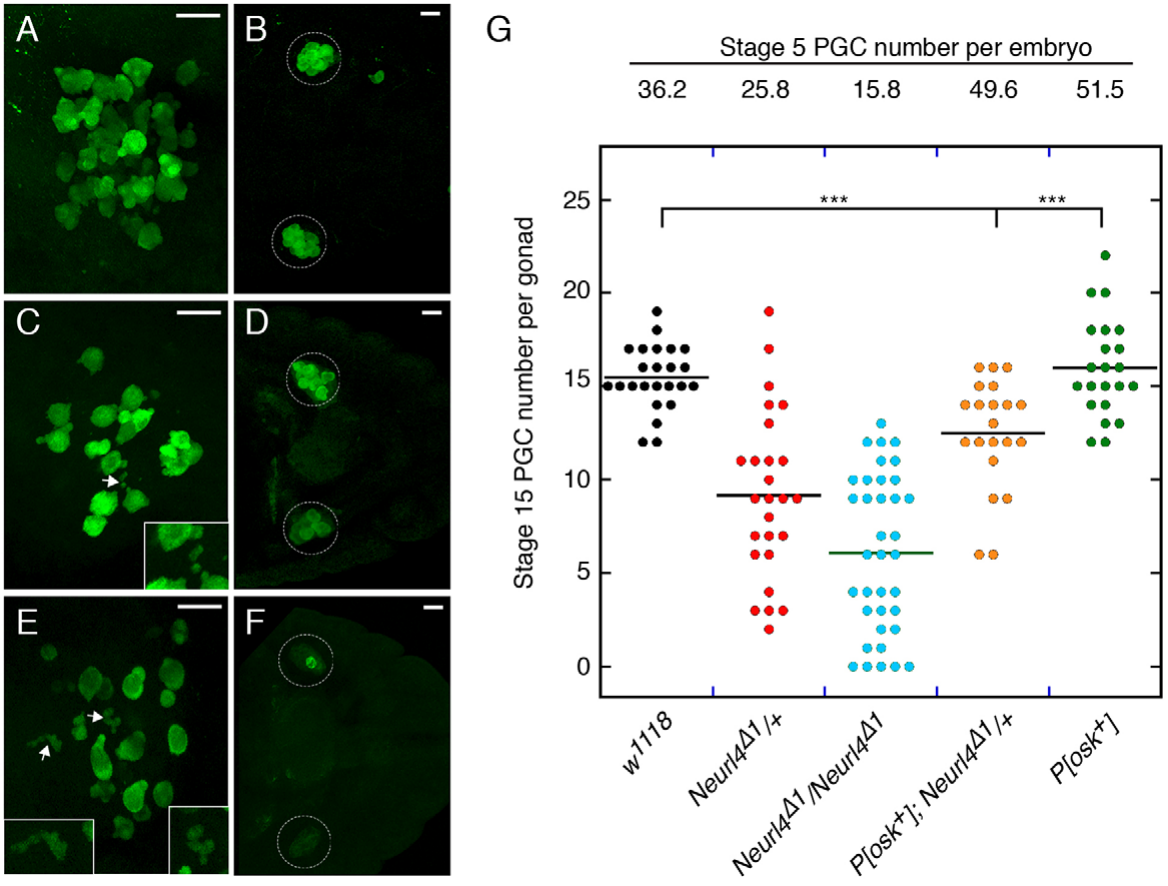
**PGC defects persist during migration and PGC numbers decline.** (A-F) All panels are stereo projections of a series of confocal sections to display all of the PGCs, detected by Vas staining (green), in each stage 10 (A,C,E) or stage 15 (B,D,F) embryo. Embryos are from mothers that are wild type (A,B), *Neurl4*^Δ*1*^/+ (C,D), or *Neurl4*^Δ*1*^/*Neurl4*^Δ*1*^(E,F). Migrating PGCs of stage 10 wild type embryos are now irregular in shape with small projections, but generally similar in size. The PGCs of embryos from mothers with reduced *Neurl4* activity can be greatly elongated and considerably misshapen (arrows, and insets in C and E). At stage 15 the PGCs have coalesced with the somatic cells of the gonad. Reduced maternal *Neurl4* activity leads to fewer PGCs in the gonads, so that in some embryos from *Neurl4*^Δ*1*^/*Neurl4*^Δ*1*^ mothers, there are few or no PGCs in the gonads (circles). Scale bars in all panels are 20 µm. (G) Number of PGCs at stages 5 and 15. The loss of PGCs at the later stage is not simply due to fewer initial PGCs, as increasing PGC number by overexpression of Osk to greater than wild type does not restore normal PGC number at stage 15. *P* values were derived from unpaired two-tailed Student's *t*-test. ***P<0.001.

The PGC defects observed in *Neurl4* mutant embryos prior to gastrulation could be a transient defect, or they might persist during migration. To address this issue we monitored PGCs at stage 10, midway through migration. Fig. 2 shows stereo projections of a series of confocal images to display all of the PGCs in individual stage 10 embryos. The migrating PGCs of wild type embryos were somewhat irregular in shape with large protrusions (Fig. 2A). In the *Neurl4* mutants (Fig. 2C,E), multiple defects were observed: the protrusions were often smaller than normal; there were displaced vesicles just as seen in stage 5 embryos; and the PGCs could be elongated relative to those in wild type embryos (compare Fig. 2C,E to 2A).

### Altered PGC morphology is not due to apoptotic membrane blebbing

Characteristic features of apoptosis include membrane blebbing and the formation of apoptotic bodies (Mills et al., 1998; Wyllie et al., 1980). Because PGCs in *Neurl4* mutant embryos shared these features, apoptosis might be the underlying cause. To test this interpretation, mutant embryos were stained for Caspase-3, a marker of apoptosis (Srinivasan et al., 1998). Neither wild type nor *Neurl4*^Δ*1*^*/+* stage 5 embryo PGCs were positive for Caspase-3 (supplementary material Fig. S1A,B). By contrast, Caspase-3 was readily detectable in stage 5 embryos in which apoptosis was induced by overexpression of *hid* (Grether et al., 1995) (supplementary material Fig. S1C). These results argue that the PGC phenotype of *Neurl4* mutant embryos was not a consequence of apoptosis.

### Isoprenylation is required for the abnormal PGC phenotype

**Fig. 3.**
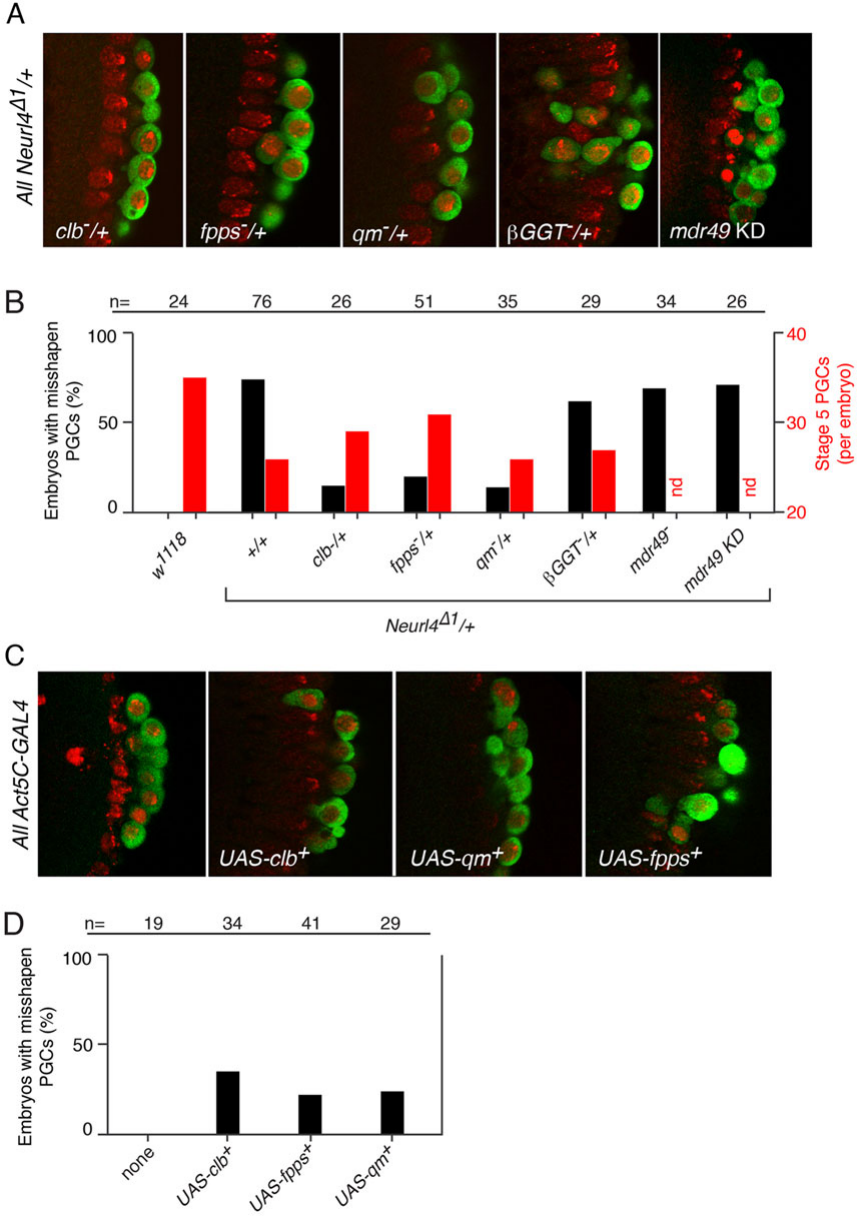
**The PGC phenotype can be suppressed or induced by altering activity of the isoprenoid biosynthetic pathway.** (A) Stage 5 embryos (posterior region) from mothers heterozygous for *Neurl4*^Δ*1*^ and also heterozygous for *clb^11.5^*, *fpps^K06103^*, *qm^L14.4^*, or *βGGT^xs-2554^*, or with *mdr49* KD, as indicated. For all panels in the figure PGCs are detected by Vas staining (green), and DNA by TO-PRO-3 (red). (B) Frequency of PGC phenotypes. The maternal genotypes are shown at bottom, except for *mdr49*^-^ and *mdr49* KD. The *mdr49*^−^ embryos were from *mdr49/CyO* mothers, and were *mdr49^3.16^/mdr49^3.16^*. The *mdr49 KD* embryos had the *TRiP-mdr49* transgene and *Act5C-GAL4*. The percentage of embryos with abnormal PGCs (as seen in the examples in A) is indicated by black bars according to the scale at left. The total number of PGCs per embryo is indicated by red bars according to the scale at right (nd, not determined). (C) Stage 5 embryos (posterior region) expressing, under *Act5C-GAL4* control, UAS transgenes as indicated. The PGC defects are typically not as strong as from reduced *Neurl4* activity, but PGCs with buds or irregular shapes are often present when the UAS transgenes are present. (D) Frequency of PGC phenotypes (examples in C) in embryos with *Act5C-GAL4* and the transgene indicated at bottom. *n* values in B and D are for the number of embryos scored.

One explanation for the morphological abnormalities of PGCs in *Neurl4* mutants is that migration was initiated inappropriately, with the PGCs responding to signals promoting migration. We reasoned that altering the activity of genes required for PGC migration might affect the *Neurl4* PGC phenotype. Four such genes, *clb*, *qm*, *fpps* and *βGGT*, encode proteins that function in isoprenoid biosynthesis and are thought to contribute to migration by geranylation of the chemoattractant (Santos and Lehmann, 2004). Females heterozygous for both *Neurl4*^Δ*1*^ and a mutant allele of one of these genes were crossed with wild type males, and stage 5 progeny embryos scored for PGC defects. Reducing the maternal contribution for *clb*, *qm* or *fpps* caused a dramatic suppression of the *Neurl4* PGC phenotype (Fig. 3A,B). Conversely, overexpression of *clb*, *qm* or *fpps* in *Neurl4^+^* embryos led to phenotypes similar to those caused by reduction of *Neurl4* activity (Fig. 3C,D). Why the *βGGT* mutant did not suppress the *Neurl4* phenotype is not known, but the encoded protein might be present at a high enough level that removing one copy of the gene was not enough to cause an effect.

The isoprenoid biosynthesis pathway is required for farnesylation and geranylation of multiple proteins, not only the chemoattractant for PGC migration (Zhang and Casey, 1996). These lipid modifications can facilitate attachment of proteins to cell membranes and are often essential for function of the proteins. An unusual feature of the chemoattractant, which sets it apart from the other modified proteins which are associated with membranes, is dependence on an export pathway requiring the ATP-binding cassette (ABC) transporter encoded by the *mdr49* gene (Ricardo and Lehmann, 2009). Notably, mutation of *mdr49* did not affect the *Neurl4* phenotype (Fig. 3B). This analysis made use of heterozygous mutant mothers to generate *mdr49* mutant embryos. In principle, maternal *mdr49* could have been sufficient for function, explaining the lack of suppression. However, transcript analysis from modENCODE shows no detectable *mdr49* mRNA in ovaries (http://flybase.org/reports/FBgn0004512.html). Furthermore, KD of *mdr49* in the embryo also had no effect on the *Neurl4* PGC phenotype (Fig. 3A,B). Therefore, suppression of the *Neurl4* mutant phenotype by reduced isoprenoid biosynthetic activity was presumably due to reduced activity of one or more of the many proteins whose association with the membrane relies on farnesylation or geranylation.

### Neurl4 is a centrosomal protein

**Fig. 4.**
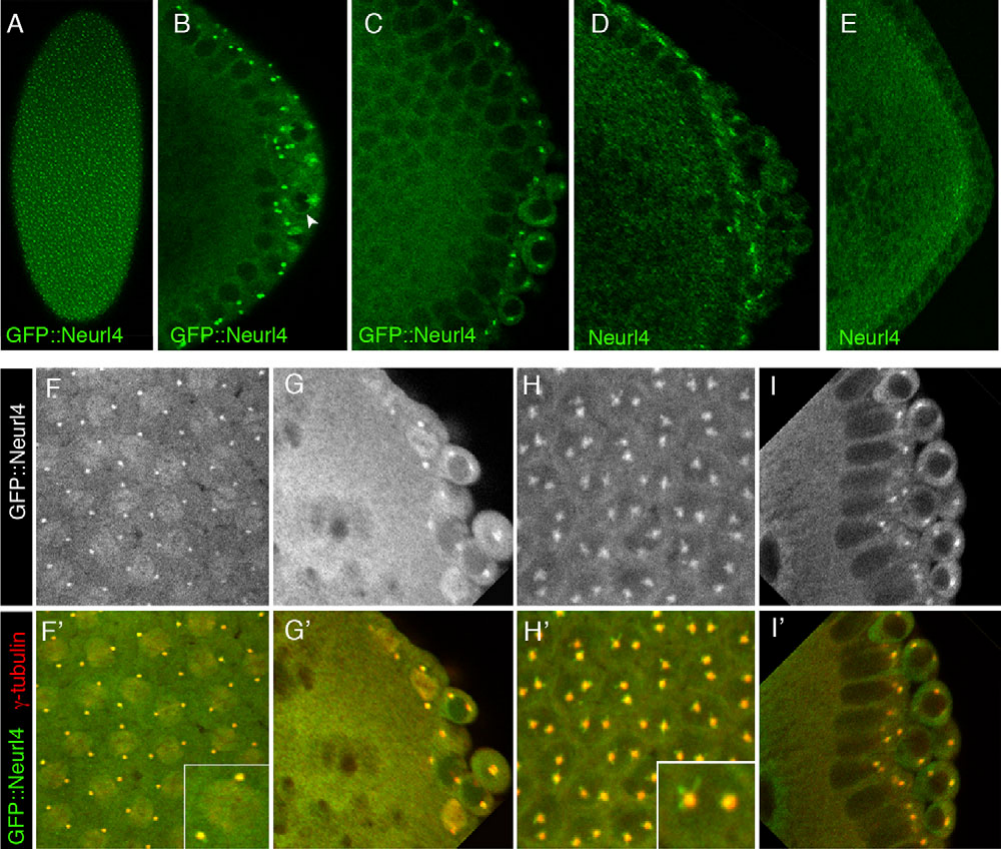
**Subcellular distribution of Neurl4.** (A-C) GFP::Neurl4 in stage 5 embryos from mothers with *P[UAS-GFP::Neurl4]/P[nos-gal4-vp16]*. Panel A is an early embryo, posterior at bottom. The confocal section is close to the apical surface and shows the foci of GFP::Neurl4. B and C show sections through embryos, including only the posterior region with PGCs at right. Apical foci of GFP::Neurl4 are visible in both panels. In B the foci in the PGCs are star-shaped, while in C they are smaller and more regular. This variability is also seen in the foci associated with somatic nuclei (below). Perinuclear enrichment is indicated by an arrowhead. (D-E) Neurl4 detected with anti-Neurl4 antibodies. D is wild type, and E is from a *Neurl4*^Δ*1*^*/Df(3L)ED4543* mother. The staining pattern in wild type is similar to GFP::Neurl4, and is largely absent in the mutant. (F-I′) Paired panels show GFP::Neurl4 alone (upper panels F-I) or both GFP::Neurl4 (green) and γ-tubulin (red) (lower panels F′-I′). (F,F′) Somatic cells of the syncytial blastoderm embryo, showing GFP::Neurl4 colocalized to foci (centrosomes) with γ-tubulin. The inset is a higher magnification view of a part of the image. (G,G′) PGCs of the syncytial blastoderm embryo showing similar colocalization of GFP::Neurl4 and γ-tubulin. (H,H′) somatic cells of the cellular blastoderm embryo, showing how the GFP::Neurl4 signal is sometimes extending away from the γ-tubulin signal (see inset). (I,I′) PGCs of the cellular blastoderm embryo.

Mammalian Neurl4 is a centrosomal protein (Al-Hakim et al., 2012; Li et al., 2012). To evaluate the distribution of Neurl4 in *Drosophila* embryos we used two approaches. The first was immunodetection with antibodies raised against recombinant Neurl4 protein (Fig. 4D,E). The specificity of the antibodies was evaluated by comparing syncytial blastoderm stage embryos from wild type or *Neurl4*^Δ*1*^*/Df(3L)ED4543* mothers. Regions of Neurl4 concentration were located apical to the somatic nuclei in wild type embryos (Fig. 4D). There was no corresponding signal in the mutant embryos, confirming the specificity of the antibodies (Fig. 4E). Neurl4 protein was also concentrated in specific foci in PGCs. The positions of these foci varied, as expected since the PGCs do not share the regular polarity of the somatic nuclei (Fig. 4D).

We also used a *GFP::Neurl4* transgene, expressed under UAS/GAL4 transcriptional control, to monitor protein distribution (Fig. 4A). The results were similar, albeit not identical to immunodetection of Neurl4. Just as for Neurl4, GFP::Neurl4 was concentrated apically, although more clearly enriched at two foci above each nucleus (Fig. 4B,C). At a lower intensity, GFP::Neurl4 was slightly enriched in a perinuclear zone in PGCs (Fig. 4B, arrowhead). The GFP::Neurl4 foci are expected to be centrosomes, based on the distribution of mammalian Neurl4. To test this prediction, GFP::Neurl4 and the centrosomal protein γ-tubulin were detected in embryos at syncytial blastoderm stage (Fig. 4F,G) and cellular blastoderm stage (Fig. 4H,I): at both stages GFP::Neurl4 colocalized with γ-tubulin in the bright foci, confirming that they are centrosomes. The distribution of GFP::Neurl4 in centrosomes was somewhat variable, being either tightly colocalized with γ-tubulin, or most enriched in the central zone but also spreading away in rays. Examples of this can be seen in PGCs in Fig. 4B and in somatic cells in Fig. 4H. Why the patterns of Neurl4 and GFP::Neurl4 were not quite identical is uncertain. One possibility is that Neurl4, like its mammalian homolog, is regulated by proteasome degradation (Al-Hakim et al., 2012), and that the GFP fusion protein is less susceptible to turnover.

### Neurl4 downregulates CP110 to prevent PGC defects

In cultured mammalian cells Neurl4 is implicated in preventing formation of ectopic microtubule organizing centers (Li et al., 2012) and in the regulation of centrosome architecture (Al-Hakim et al., 2012). These effects are achieved, at least in part, by reducing the level of the centrosomal protein CP110 by ubiquitylation. We asked if *Drosophila* Neurl4 also acts in downregulation of CP110.

**Fig. 5.**
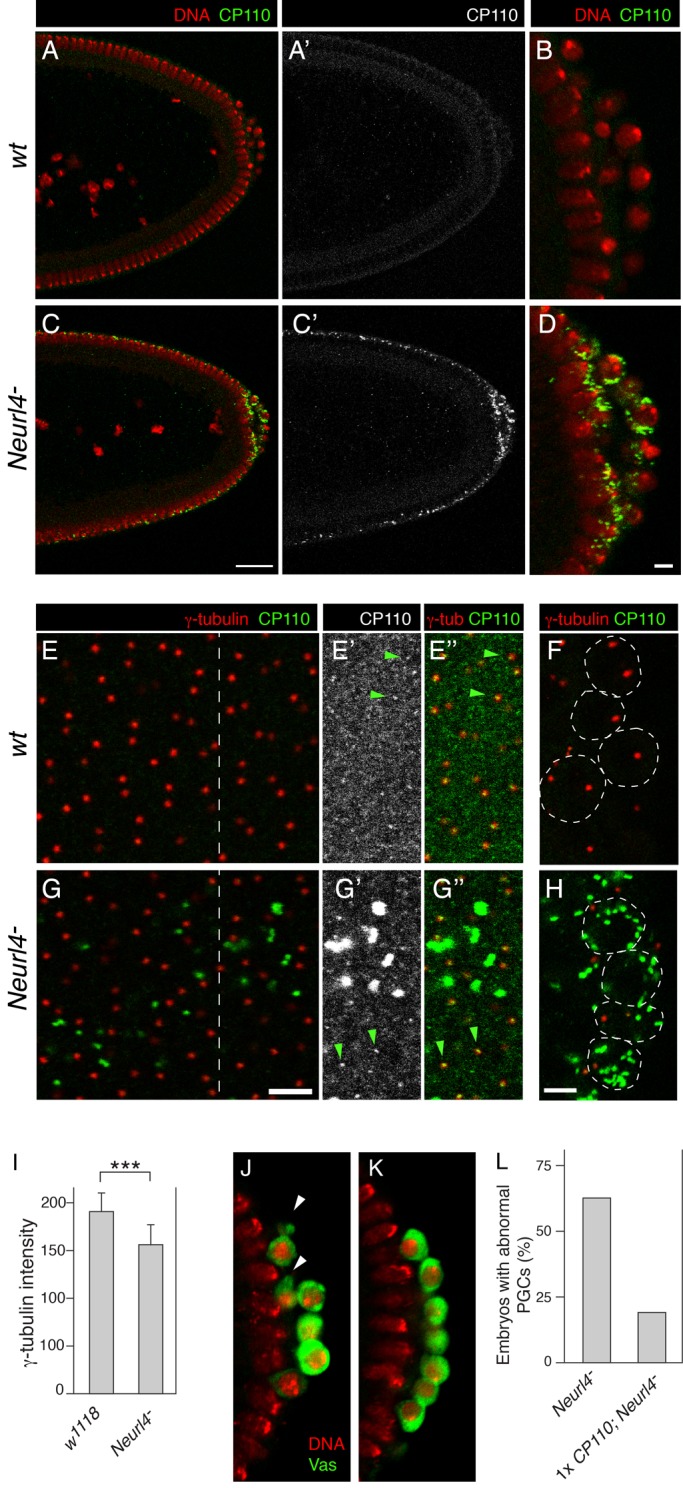
***Neurl4* downregulates CP110 to prevent PGC defects.** (A-D) Posterior portions of embryos from mothers with wild type (*w^1118^*) or reduced *Neurl4* activity, with posterior to the right. For the latter, the examples shown were from maternal KD of *Neurl4*; similar results were obtained with *Neurl4*^Δ*1*^*/Df(3L)Neurl4* mothers. The images were obtained under identical conditions, except for a higher zoom for panels B and D. A and C show both CP110 and DNA, while A′ and C′ show only CP110. In D, CP110 can be seen in the PGCs (extreme right, spherical nuclei) and closely associated with underlying somatic nuclei which have a more elongate appearance. Scale bars are 30 µm (A,C) and 5 µm (B,D). (E-H) Comparison of CP110 and γ-tubulin in embryos from mothers with wild type (*w^1118^*) or reduced *Neurl4* activity. For the latter, the examples shown were from *Neurl4*^Δ*1*^*/Df(3L)Neurl4* mothers; similar results were obtained with maternal KD of *Neurl4*. The raw confocal images were obtained under identical conditions, except for a higher zoom for panels F and H. Panels E and G are sections parallel to the surface of the central region of the embryos, in the apical region containing centrosomes apical to nuclei. The portion of each image to the right of the dashed line is shown again (E′,E″,G′,G″), following identical adjustments to the green channel to reveal low intensity signals. For E′ and G′, only the CP110 channel is shown, while in E″ and G″ both CP110 and γ-tubulin channels are shown (no adjustment to the γ-tubulin channel). Examples of CP110 foci that overlap with γ-tubulin are indicated by green arrowheads. Panels F and H are sections through PGCs, some of which are outlined by dashed lines. Scale bars are 5 µm. (I) Quantitation of γ-tubulin fluorescence intensity in centrosomes from embryos as in E and G. Stacked z series images were focused and maximum intensities measured, all in ImageJ. *P* values were derived from unpaired two-tailed Student's *t*-test. ***P<0.001. (J-L) Suppression of the *Neurl4* PGC phenotype by reducing dosage of *CP110*. J and K are examples of stage 5 embryos (posterior region) with PGCs detected by anti-Vas (green), with DNA (TO-PRO-3) in red. Maternal genotypes for the embryos are (J) *FM7c/+; Df(3L)Neurl4/+* and (K) *Df(1)Excel6255/+; Df(3L)Neurl4/+*. The two genotypes represent sibling flies obtained from the same cross (*FM7c* is a balancer chromosome). Note that the frequency of PGC defects is not influenced by *FM7c* (compare to *Df(3L)Neurl4/+* in Fig. 1F). Arrows indicate examples of misshapen cells. Panel L shows the frequency of embryos with abnormal PGCs. For *Df(3L)Neurl4/+ n* is 26, and for *Df(1)Excel6255/+; Df(3L)Neurl4/+ n* is 31. Error bars indicate standard deviation from the mean.

Immunodetection of CP110 in stage 5 embryos revealed a dramatic difference for the *Neurl4* mutant. In wild-type (*wt*) embryos the level of CP110 was very low, effectively undetectable under the imaging conditions used for panels A-D in Fig. 5. By contrast, in the *Neurl4* mutant embryos CP110 appeared in bright foci which were usually apical to the nuclei (Fig. 5C). At the posterior, there was a higher density of the foci, with CP110 enriched in the PGCs. This enrichment was region-specific, rather than PGC-specific, since the foci were also more abundant outside the PGCs close to the somatic nuclei in the same region (Fig. 5D).

Although CP110 is normally associated with centrosomes, the CP110 foci in *Neurl4* mutant embryos did not show the stereotypical centrosome pattern of two foci per cell (or somatic nucleus): there were multiple foci per cell/nucleus in the posterior region, and what appeared to be a variable number elsewhere. To characterize the relative positions of centrosomes and the CP110 foci, embryos were stained for both CP110 and γ-tubulin, and viewed in a focal plane parallel to the surface of the embryo (Fig. 5E,G). Under imaging conditions similar to those of Fig. 5A-D, CP110 was detected only in the mutant embryos, and the foci were distinct from γ-tubulin. Although the distribution of γ-tubulin was normal in the mutant embryos, the level of γ-tubulin appeared lower (Fig. 5G), independent of the variation in intensity expected in a single confocal section in which not all of the centrosomes will be centered precisely in the focal plane. To avoid the variation due to focal plane position, and to more rigorously test γ-tubulin levels in the centrosomes, stacks of z section images were focused and signal intensities measured. The *Neurl4* mutants had a small but significant decrease in γ-tubulin levels (Fig. 5I). Within the PGCs the CP110 foci were also distinct from centrosomes. Because PGCs do not have the regular polarity of the blastoderm nuclei, the position of the centrosomes varied among different PGCs, and 0, 1 or 2 centrosomes appeared in a particular focal plane. Nevertheless, the many foci of CP110 in the mutant PGCs did not overlap with the γ-tubulin foci (Fig. 5H). Just as elsewhere in the embryo, the level of γ-tubulin in the PGC centrosomes appeared lower (Fig. 5F,H).

In addition to the bright foci of CP110 seen in the *Neurl4* mutant embryos, a dispersed granular staining pattern was detected in both wild type and mutant embryos using higher sensitivity for imaging. There was a slight enrichment of CP110 signal at positions showing colocalization with γ-tubulin and thus corresponding the centrosomes (Fig. 5E,G, green arrowheads). CP110 signal intensity in the centrosomes appeared to be higher in the *Neurl4* mutant, reminiscent of the effect of depleting Neurl4 in mammalian cells (which do not have the intense extracentrosomal CP110 foci we describe here). However, quantitation of this effect is difficult, given the modest differences in CP110 signal in, and away from, the centrosomes.

Reasoning that CP110 enrichment in centrosomes might be stronger in a different cell type, we also examined the distribution of the protein in the layer of follicle cells that surround the oocyte. Detection of γ-tubulin and CP110 in these cells during the mid stages of oogenesis revealed a pattern more like that reported in cultured mammalian cells, with prominent CP110 foci which usually overlapped with, or were close to, foci of γ-tubulin (supplementary material Fig. S2A-C). In *Neurl4* mutant ovaries the same pattern persisted: the foci remained mostly or entirely coincident with centrosomes. Because the CP110 signal intensity in the foci was substantially higher than in the surrounding area (unlike the situation in early embryos), the conclusion that CP110 was normally associated with centrosomes can be made with more confidence. In addition, comparison of CP110 signal intensity in centrosomes between wild type and mutant samples confirmed a small yet significant increase in the mutant (supplementary material Fig. S2D-F), much as observed in mammalian cells.

Our results revealed two effects of loss of *Neurl4* activity on CP110: a modest enhancement of the protein in centrosomes, which may be common to a wide range of cell types; and the appearance of ectopic foci distinct from centrosomes and with a much higher level of CP110. The latter effect was not universal, and even in the early embryo was clearly more pronounced in a narrow posterior domain which includes the PGCs. Because the strongest effect of the *Neurl4* mutant on CP110 was precisely where cells misbehave, it seemed likely that elevated CP110 levels might be responsible for the abnormal PGCs. If so, that phenotype might be suppressed by lowering *CP110* gene dosage. Examination of embryos from females heterozygous for a *Neurl4* mutation and with only one copy of the *CP110* gene revealed a 3 fold reduction in the fraction of embryos with abnormal PGCs, as compared to the *Neurl4* mutant alone (Fig. 5J-L).

### *Neurl4* has an additional role in PGC formation

**Fig. 6.**
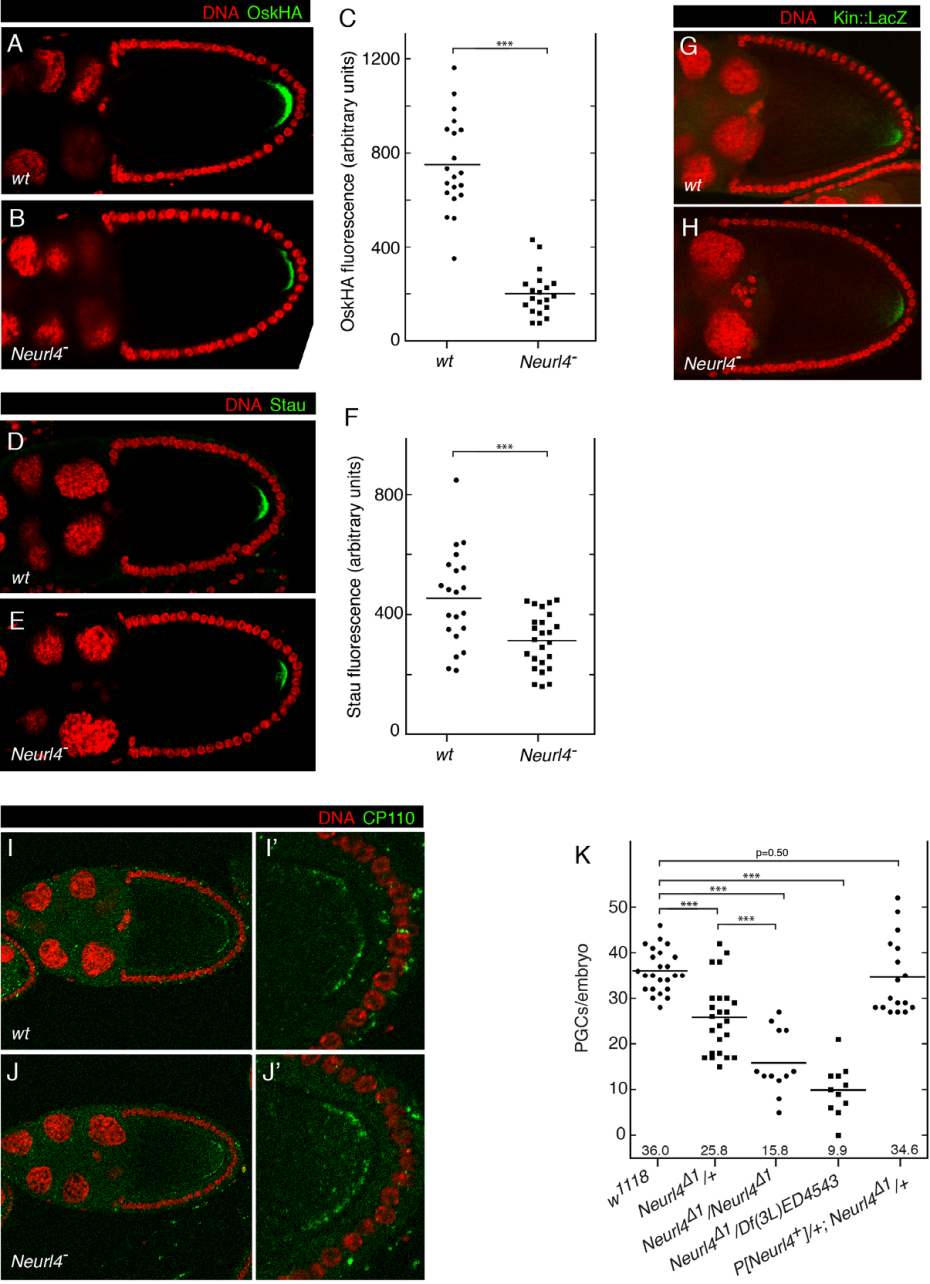
***Neurl4* contributes to Osk protein expression and PGC formation.** (A-C) OskHA expression in oocytes with (A) wild type (w1118) or (B) reduced *Neurl4* activity (the examples shown were from maternal KD of *Neurl4*; similar results were obtained with *Neurl4*^Δ*1*^*/Df(3L)Neurl4* mothers). Imaging conditions were the same for both. (C) Fluorescence levels were quantitated in FIJI, measuring total signal intensity within the posterior crescents. *P* values were derived from unpaired two-tailed Student's *t*-test. ***P<0.001. (D-F) Stau expression in in oocytes with (D) wild type (w1118) or (E) reduced *Neurl4* activity as in A and B. Imaging conditions were the same for both. (F) Fluorescence levels were quantitated in FIJI, measuring total signal intensity within the posterior crescents. *P* values were derived from unpaired two-tailed Student's *t*-test. ***P<0.001. (G-H) Detection of Kin:LacZ in (G) wild type (*w^1118^*) and (H) *Neurl4* mutant (*Neurl4*^Δ*1*^*/Neurl4*^Δ*1*^) oocytes. (I-J) CP110 in (I) wild type (*w^1118^*) and (J) *Neurl4* mutant (*Neurl4*^Δ*1*^*/Df(3L)Neurl*) stage 10 egg chambers. The panels at right (I′,J′) are higher magnification views to show the enriched cortical CP110 at the posterior of the oocytes. (K) Numbers of PGCs for stage 5 embryos from females of the genotypes shown below the chart. Averages are indicated by horizontal lines and shown beneath the dot plots. *P* values were derived from unpaired two-tailed Student's *t*-test. ***P<0.001. Note that the PGC number phenotype becomes stronger as *Neurl4* activity decreases. This is unlike the abnormal PGC phenotype, which is similar for heterozygous and homozygous or hemizygous *Neurl4* mutants (see Fig. 1).

*Neurl4* is not an essential gene, as homozygous or hemizygous mutants were viable and appeared healthy. However, a fraction of the progeny of *Neurl4* mutant mothers were agametic and thus infertile (supplementary material Table S1). Examination of embryos from *Neurl4* mutant mothers revealed that, in addition to the abnormal PGC morphology, the number of PGCs was reduced. At stage 5, embryos from wild type females had an average of 36 PGCs. Reducing maternal *Neurl4* activity led to a decrease in average number of PGCs (Fig. 6K). Not surprisingly, there were also fewer PGCs at a later stage of embryonic development (Fig. 2). The *Neurl4^+^* transgene restored the number of PGCs to wild type levels (Fig. 6K), confirming that the phenotype was due to reduced *Neurl4* activity.

Although some loss of PGCs might result from their abnormal morphology, it seemed likely that the initial number of PGCs was lower in *Neurl4* mutant embryos. Consistent with this interpretation, while the PGC morphology defect could be strongly suppressed by reducing the dosage of isoprenoid biosynthesis genes, suppression of the PGC number defect in stage 5 embryos was much weaker (Fig. 3B).

PGCs derive from polar plasm, which is assembled at the posterior of the oocyte during oogenesis. The pathway of polar plasm assembly involves the initial localization of *osk* mRNA to the posterior pole of the oocyte. After translation at this site, Osk protein recruits other required factors. How much Osk is present dictates the amount of polar plasm to be assembled and the number of PGCs formed (Ephrussi and Lehmann, 1992; Smith et al., 1992). Thus, one explanation of the *Neurl4* PGC number defect is a deficiency in Osk protein accumulation. Initial immunostaining tests revealed reduced Osk levels. To facilitate quantitation (the anti-Osk antibodies have substantial background staining) we used an *osk::HA* transgene, which expressed *osk* under its normal transcriptional control and fully rescues an *osk* mutant (Materials and Methods) (Kim et al., 2015). At stage 10 of oogenesis Osk::HA was strongly expressed in wild type, but levels were substantially lower for the *Neurl4* mutant (Fig. 6A-C).

The reduced levels of Osk in *Neurl4* mutant oocytes should lead to a reduction in the number of PGCs formed, and this raises the question of whether reduced PGC number can be attributed entirely to lower Osk, or if the morphological defects of mutant PGCs also contribute to their loss. To address this question we increased the initial number of PGC by overexpression of Osk (Smith et al., 1992). However, despite having substantially more PGCs at stage 5, by stage 15 the number of PGCs in embryos from *Neurl4* mothers was significantly lower than for wild type (Fig. 2G).

Low Osk levels have several possible origins, including impaired translation or localization of *osk* mRNA. To test for a defect in *osk* mRNA localization, mutant ovaries were stained for Stau protein, which associates with *osk* mRNA and faithfully reveals its distribution in ovaries (St Johnston et al., 1991). Stau was consistently present at the posterior of stage 10 oocytes, both wild type and *Neurl4* mutant, but the level was lower in the mutant oocytes (Fig. 6D-F). Localization of *osk* mRNA relies on microtubule-dependent movements, and correct organization of microtubules is essential (St Johnston, 2005). The *Neurl4* mutants did not display gross defects in microtubule organization in the oocyte, since localization of the microtubule polarity marker Kin:LacZ (Clark et al., 1994) was normal in *Neurl4*^Δ*1*^*/Neurl4*^Δ*1*^ egg chambers (Fig. 6G-H). Given the involvement of CP110 in the PGC phenotype, and the importance of microtubules in *osk* mRNA localization, we looked for changes in CP110 that might affect microtubule organization. CP110 was slightly enriched in a posterior cortical region of the oocyte at the time when *osk* mRNA is undergoing localization (Fig. 6I), but there was no substantial change in this pattern or level in *Neurl4* mutants (Fig. 6J).

## DISCUSSION

Prior analysis of Neurl4 protein, from studies with cultured mammalian cells, demonstrated its association with centrosomes and identified functions in control of centrosome organization (Al-Hakim et al., 2012; Li et al., 2012). The action of Neurl4 was associated with a specific biochemical activity, downregulation of centrosomal protein CP110 by ubiquitylation (Li et al., 2012). We found that reducing *Neurl4* activity also led to elevated CP110 levels in *Drosophila*, but the severity and type of the defect varied dramatically depending on cell type. In mammalian cells the effect on CP110 is to increase its concentration in centrosomes. We found the same effect in an ovarian tissue, the layer of follicle cells that surround the oocyte. Although harder to quantify, this change also appeared to occur in the centrosomes of the blastoderm stage embryo, where centrosomes also displayed a modest decrease in γ-tubulin. The more striking change was observed only in the embryo: the appearance of high levels of CP110 in ectopic foci distinct from centrosomes. For most of the cells in the embryo, there appeared to be no adverse effects from the elevated CP110. This is consistent with work characterizing *Drosophila* CP110, in which CP110::GFP fusion proteins were overexpressed in flies with no overt effect on viability or fertility. Likewise, deleting the *CP110* gene did not affect viability or fertility. Instead, the level of CP110 subtly influences centriole length: slightly longer in the absence of CP110, and slightly shorter when CP110 is overexpressed (Franz et al., 2013).

Although reduced *Neurl4* activity induced ectopic CP110 foci throughout the embryo, a narrow posterior region was most strongly affected. CP110 was also enriched cortically at the posterior of the oocyte, and this could contribute to the later embryonic defects. The posterior region of the embryo with the most ectopic CP110 foci included the PGCs as well as the underlying layer of somatic nuclei, but it was only the PGCs for which obvious changes in cell behavior were detected. The abnormal morphology of the PGCs was indeed caused by elevated CP110, as this phenotype could be partially suppressed by reducing dosage of the *CP110* gene. Our results did not reveal whether the phenotype resulted from the weak enhancement of CP110 in centrosomes, or the far more dramatic formation of multiple ectopic foci of CP110, although the latter seems more likely simply because of the greater deviation from wild type. Notably, only a subset of the PGCs had abnormal morphology, even though all had elevated CP110. This suggests that the elevated CP110 created a predisposition for altered morphology, with a stochastic event or the contribution of some limiting factors or conditions then required to complete the process.

*Neurl4* mutants affect the number of PGCs, as well as their behavior. The lower number of PGCs has two causes. First, the initial formation of PGCs is constrained by reduced levels of Osk protein at the posterior of the oocyte. We have not explored this defect in detail, but it appears to be due at least in part to a reduced level of localized *osk* mRNA, the source for production of Osk. Although localization of *osk* mRNA relies on microtubules (Pokrywka and Stephenson, 1995; Brendza et al., 2002; Cha et al., 2002; Zimyanin et al., 2008), the organization of microtubules within the oocyte remains controversial. Fusion proteins containing the motor domains of Nod and kinesin localize, respectively, to the anterior and posterior regions of the oocyte, suggesting that they are marking the minus and plus ends of microtubules (Clark et al., 1994; Clark et al., 1997). However, by direct visualization the microtubules appear to be nucleated from the anterior and lateral cortical regions of the oocyte, extending in all directions to form an anterior-posterior gradient (Cha et al., 2001; MacDougall et al., 2003). Tracking of *osk* mRNA movements indicates a weak bias for posterior orientation (Zimyanin et al., 2008). We did not detect any substantial change in microtubule organization in the *Neurl4* mutant, as judged by the distribution of the Kin-lacZ fusion protein. Nevertheless, it seems possible that the posterior cortical enrichment of CP110 may influence microtubule organization in some subtle manner to facilitate polarized movements or local anchoring of *osk* mRNA, with this activity sensitive to *Neurl4*. This suggestion of an effect of *Neurl4* on microtubules is supported by the modest reduction in the level of γ-tubulin in centrosomes in *Neurl4* mutant embryos.

Independent of the initial number of PGCs in *Neurl4* mutant embryos, some are lost during embryogenesis, with some late stage embryos having few if any PGCs. The continuing loss of PGCs was confirmed in experiments in which Osk was overexpressed: despite an initial increase in the number of PGCs in *Neurl4* mutants, there were nevertheless fewer PGCs than wild type after migration to the gonads (Fig. 2). The loss of PGCs is presumably associated with their abnormal morphology and the apparent budding off of small vesicles.

The discovery of a novel PGC mutant phenotype and knowledge of required biochemical pathways provides the means for understanding aspects of PGC biology not previously addressed. A key question is whether the mammalian *Neurl4* gene plays a similar role, with more dramatic defects in germ-line cells than detected in cultured cells.

## MATERIALS AND METHODS

### Flies

*Df(3L)ED4543*, *Df(1)Exel6255*, *Df(3L)fz-GF3b*, *P[EY12221]*, *P[hs-hid]*, *fpps^K06103^*, *matalpha4-GAL-VP16*, *GAL4::VP16-nos.UTR*, *P{TRiP.GL01219}attP40* (*Neurl4* KD), *P{TRiP.HMS00400}attP2* (*mdr49* KD) and *Act5C-GAL4* were obtained from the Bloomington Stock Center. Alleles of *blue* were from Douglas Ruden. Mutants *Tre1^ΔEP5^*, *clb^11.5^*, *qm^L14.4^*, *βggt^xs2554^*, mdr49^Δ3.16^ and *wun^ce^* were from Ruth Lehmann, as were the *UAS-clb*, *UAS-fpps* and *UAS-qm* flies. *Kin:LacZ* flies were from Dave Stein. *Df(3L)Neurl4*, a 16 kb deletion affecting 6 genes – *Hsc70Cb*, *Neurl4*, *CG6833*, *CG13484*, *CG43986* and *CG32138* – was made using *PBac* (White et al., 1998) *CG32138^f0183^*^0^ and *P{XP}Hsc70Cb^d06126^* from the Bloomington Stock Center by the method of (Parks et al., 2004). Mobilization of the P element of *P[EY12221]* yielded precise excisions, as well as Neurl4^Δ1^ and Neurl4^Δ2^.

*CG6451* was previously named *blue*, reflecting the ovarian phenotype (in germ line clones) of a P element insertion chromosome. The P insertion chromosome is lethal over *Df(3L)fz-GF3b/TM6b* (which has a deletion of the 70C1-2; 70D4-5 region), supporting the view that the P insertion was responsible for lethality. Multiple EMS-induced mutants, isolated on the basis of failure to complement the lethality of the P insertion chromosome, all had the *blue* phenotype in germ line clones (Douglas Ruden, personal communication). Thus the lethality and *blue* phenotype appear to be due to mutation of the same gene. However, this gene is not *CG6451*, based on two lines of evidence. First, the *blue* EMS alleles are complemented by *Df(3L)Neurl4*, which lacks the entire *CG6451* gene. Second, the *CG6451* genomic transgene, which rescues the PGC phenotype described here, fails to rescue lethality of the *blue* mutants. Based on these results, we have named *CG6451* as *Neurl4*, to adhere to the nomenclature for the mammalian gene and to reflect its dominant phenotype. The *blue* gene presumably lies within the region deleted in *Df(3L)fz-GF3b/TM6b*, and remains unidentified.

### Transgenes

*P[Neurl4+]* contains a genomic DNA segment (3L:14036401–14044352, R5.54) inserted into a modified CaSpeR vector. *P[UAS-GFP-Neurl4]* contains the *Neurl4* coding region (with introns) and 3′ UTR fused in frame with mGFP6 (Haseloff, 1999) in the pUASp vector. The construct was expressed using a *nos-gal4-vp16* driver. The *osk::HA* transgene was made by inserting three copies of the HA epitope sequence (TACCCATACGATGTTCCTGACTATGCGGGCTAT CCCTATGACGTCCCGGACTATGCAGGATCATATCCATATGACGTTCCAGATTACGCT) after the codon for T140 in a genomic fragment of *osk* that fully rescues *osk* mutants. This places the epitope tag just after the start of Short Osk (which begins at M139). Flies with *osk::HA* as the only source of *osk* have normal patterns of Osk expression and are viable and fertile, and have been maintained in this state for several years.

### Antibodies

The SD03524 cDNA, which encodes the C-terminal 874 amino acids, was inserted into the pET15b expression vector using NarI and XhoI sites, and the protein was expressed in *E. coli* BL21 pLysS. The protein was purified by insoluble aggregate purification and used for antibody production. The protein was also transferred onto nitrocellulose strips for affinity purification of antibodies.

Antibodies were used at the following concentrations: rabbit α-Neurl4, 1:200; mouse α-γ-tubulin (GTU-88, Sigma-Aldrich), mouse anti-HA (Covance HA.11 16B12), rabbit α-Caspase-3 (BD Pharmingen), rabbit anti-CP110 (from Jordan Raff), rat α-Vasa, all 1:500; rabbit α-Staufen, 1:1000; rabbit α-Oskar, 1:3000; mouse α-LacZ, 1:100. Secondary antibodies coupled to Cy3, Cy5 or Alexa Fluor 488 (Jackson Immunoresearch Laboratories and Invitrogen) were used at 1:800, TO-PRO-3 (Invitrogen) was used at 1:1000.

### Immunodetection and imaging

Ovaries and embryos were stained as described previously (Macdonald and Struhl, 1986; Snee and Macdonald, 2004). For *P[hs-hid]* collection, embryos were collected in apple juice vials for one hour, and then heat shocked for one hour at 37°C. After one hour of recovery, the embryos were processed as usual. For detection of Neurl4, embryos were hand-peeled to remove the vitelline membrane. This method avoids exposure to methanol, to which the Neurl4 epitope(s) is sensitive. Samples were mounted in Vectashield medium (Vector Labs) and imaged using a Leica TCS-SP confocal microscope.

For analysis of PGC number and defects, a confocal z series was obtained for each embryo. The image stacks were used for PGC counts and to score for abnormal PGCs. In cases when PCG counts were not determined, embryos were examined for abnormal PGCs during imaging. Quantitation of fluorescence was done as described in the legend for each experiment, using ImageJ (Wayne Rasbad), Fiji (Schindelin et al., 2012) or Macnification (Orbicule, Inc.) with samples that were fixed, processed, and imaged in parallel.

## Supplementary Material

Supplementary Material
