# Supplementary material for: Neurl4 contributes to germ cell formation and integrity in Drosophila
Source: Biol Open. 2015 Jun 26;4(8):937–46. doi: 10.1242/bio.012351 (PMC4542285; doi:10.1242/bio.012351)
Supplement: Supplementary Material [file supp_4_8_937__index.html]

Neurl4 contributes to germ cell formation and integrity in Drosophila — Neurl4 contributes to germ cell formation and integrity in Drosophila — Supplementary Material 

# Neurl4 contributes to germ cell formation and integrity in *Drosophila*

## BIO012351 Supplementary Material

- Supplementary Material
