# Supplementary material for: Neurl4 contributes to germ cell formation and integrity in Drosophila
Source: Biol Open. 2015 Jun 26;4(8):937–46. doi: 10.1242/bio.012351 (PMC4542285; doi:10.1242/bio.012351)
Supplement: Supplementary Material [file supp_bio.012351_BIO012351supp.pdf]

Supplementary material Table S1.

Table S1. Ovary number in female progeny of mothers with reduced *Neurl4* activity.

Genotype of mother	Two ovaries	One ovary	No ovaries	n
Df(3L)fz-GF3b/+	78%	22%	0%	23
Neurl4$^{\Delta 1}$*/+*	100%	0%	0%	22
Neurl4$^{\Delta 1}$*/Neurl4*$^{\Delta 1}$	79%	16%	5%	19
Neurl4$^{\Delta 1}$*/Df(3L)fz-GF3b*	67%	22%	11%	18

Supplementary material Figures

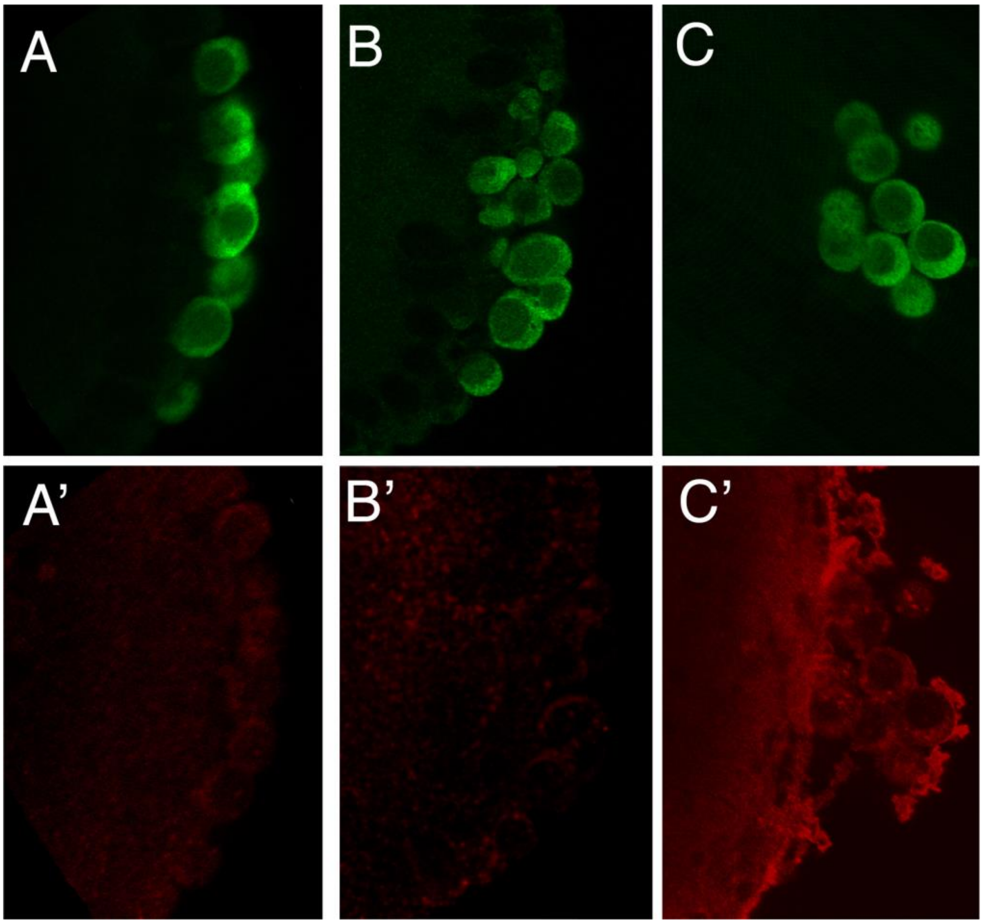

Fig. S1. Misshapen PGCs are not Caspase-3 positive. A-C. Paired panels (e.g. A and A’) show Vasa (green) and Caspase-3 (red) staining of embryos from wildtype (A, A’), *Neurl4*$^{\Delta 1}$/+ (B, B’), and *P[hs-hid]/+* (C,C’) mothers (the last genotype produces embryos with and without the *hs-hid* transgene; the embryo in the figure includes the transgene). A heat shock was used to induce *hid* expression (Methods).

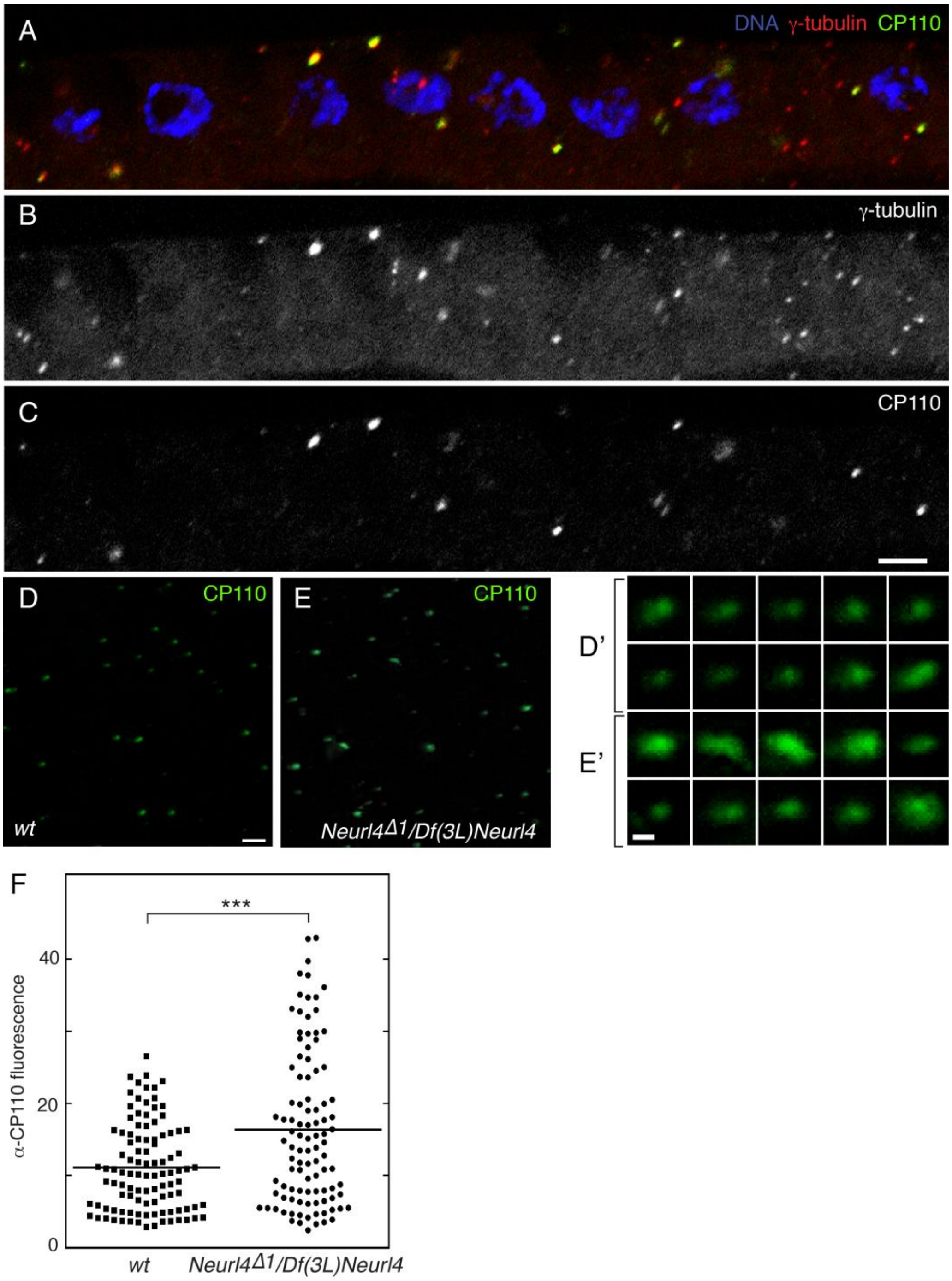

Fig. S2. Neurl4 downregulates centrosomal CP110. (A-C). Cross section of a region of the follicle cell layer surrounding the oocyte of a stage 10 egg chamber, with detection of DNA (blue), γ-tubulin (red) and CP110 (green) in A. Individual signals for γ-tubulin (B) and CP110 (C) are shown in the lower panels. Most, if not all, of the CP110 foci overlap with γ-tubulin. Scale bar is 5 µm.

(D-E). CP110 foci in the follicle cell layer of stage 10 w^{1118} (D) or *Neurl4*$^{\Delta 1}$*/Df(3L)Neurl4* egg chambers. Each image was obtained by collecting a z series stack of confocal slices extending through the follicle cell layer, from the surface of the egg chamber into the oocyte, and focusing them into a single image in Macnification. Imaging conditions were the same for both samples. Scale bar is 5 µm. For D' and E', individual foci from D and E, respectively, are shown at higher magnification (scale bar is 1 µm).

(F). Quantitation of fluorescence intensity in CP110 foci using Macnification. Total signal intensity in individual foci from the stacked and focused images was measured. P values were derived from unpaired two-tailed Student's *t*-test. ***, p<0.001.
